# Supplementary material for: Glucose Supplementation Enhances the Bactericidal Effect of Penicillin and Gentamicin on Streptococcus sanguinis Persisters
Source: Antibiotics (Basel). 2025 Jan 5;14(1):36. doi: 10.3390/antibiotics14010036 (PMC11762801; doi:10.3390/antibiotics14010036)
Supplement: Supplementary file 1 [file antibiotics-14-00036-s001.zip › antibiotics-3353739-supplementary.pdf]

SUPORTING INFORMATION

Glucose supplementation enhances the bactericidal effect of penicillin and gentamicin on *Streptococcus sanguinis* persisters

Kazuya Takada [1,2], Yoshie Yoshioka [1], Kazumasa Morikawa [2], Wataru Ariyoshi [1], Ryota Yamasaki [1, 3*]

[1]Division of Infections and Molecular Biology, Department of Health Promotion, Kyushu Dental University, Kitakyushu, Fukuoka 803-8580 Japan

[2] Division of Oral Functional Development, Department of Health Promotion, Kyushu Dental University, Kitakyushu, Fukuoka 803-8580 Japan

[3] Collaborative Research Centre for Green Materials on Environmental Technology, Kyushu Institute of Technology, 1-1 Sensui-chou, Tobata-ku, Kitakyushu, Fukuoka 804-8550, Japan

*For correspondence. E-mail r18yamasaki@fa.kyu-dent.ac.jp

Tel. (+)81 93-285-3051

Keywords: *Streptococcus sanguinis*; persister; infective endocarditis

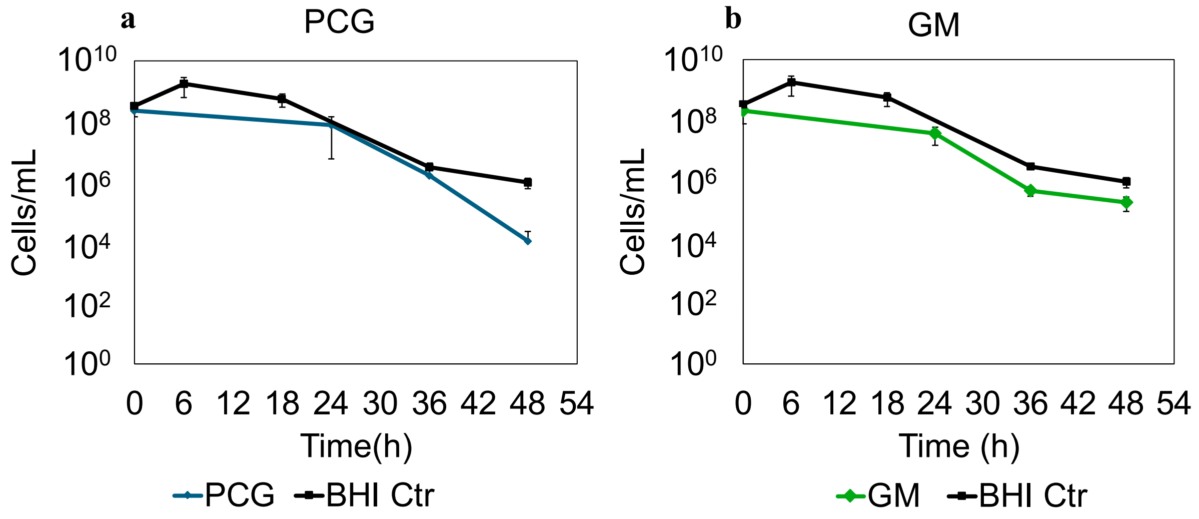

Supplemental Figure S1. Bactericidal effect of PCG (1600 µg/ml) (a) and GM (64 µg/ml) (b) treatment against *S. sanguinis* in the exponential phase. CFU were measured at each time. Error bars indicate the standard deviation across at least three biological replicates.

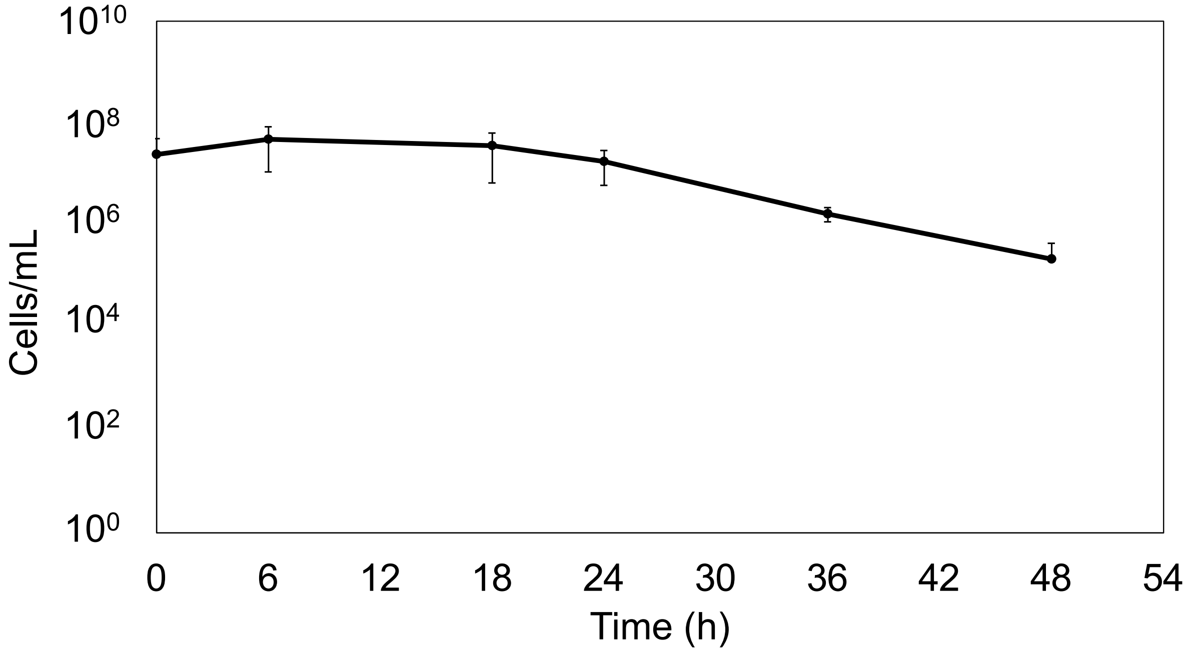

Supplemental Figure S2. Growth curve of *S. sanguinis* cultured in M9 minimal medium with glucose added to reach 100 mg/ml. CFU were measured at 0, 6, 18, 24, 36, and 48 hours. Error bars indicate the standard deviation across at least three biological replicates.
